# Assessment of Additional Risk Factors for Cardiovascular Disease and Awareness Among Adult Patients With Diabetes Mellitus: A Cross-Sectional Study From Northern Sri Lanka

**DOI:** 10.7759/cureus.30047

**Published:** 2022-10-07

**Authors:** Printhiny Manoharan, Rajeshkannan Nadarajah, Navaneethakrishnan Suganthan

**Affiliations:** 1 Medicine, District General Hospital, Kilinochchi, LKA; 2 Family Medicine, Civic Park Medical Centre, Sydney, AUS; 3 Medicine, University of Jaffna, Jaffna, LKA; 4 Medicine, Teaching Hospital Jaffna, Jaffna, LKA

**Keywords:** physician inertia, life style modifications, northern sri lanka, cardiovascular risk, diabetes

## Abstract

Introduction

Cardiovascular diseases are the leading cause of death in people with type 2 diabetes. The assessment of additional risk factors for cardiovascular disease among diabetes patients and comparing current practices with the best practices can improve patient care. The aim of this study was to assess these additional risk factors and awareness of them among adult patients with diabetes mellitus.

Materials and methods

This cross-sectional study was conducted in the general medical wards at District General Hospital Kilinochchi from June 2021 through August 2021 and included 421 patients. The data were analyzed using SPSS version 28.

Results

Most patients (70.1%) were women, and their mean age was 57.4 years. Their lifestyle-related risk factors included being overweight (9%) or obese (2.1%), smoking (8.8%), consuming alcohol (2.4%), insufficient physical activity (23.5%), and not meeting the Sri Lankan dietary guidelines for the consumption of fruits and vegetable (75.3%). In addition, 3.3% were suffering from chronic kidney disease, 6.2% from micro-albuminuria, 49.4% from hypertension, and 67.7% from hypercholesterolemia. Further, 11.4% (CI: 8.6-11.7%) had uncontrolled diabetes, only 40.1% had low-density lipoprotein (LDL) levels within the target range, and only 16.2% had systolic blood pressure within the target range.

Conclusions

The findings presented here indicate the existence of significant gaps similar to those found in the literature regarding lifestyle modifications and recommended practices for reducing the risk of cardiovascular disease. As a result, it may be necessary to address physicians’ inertia in regard to the implementation of best practices, and there is a clear need to educate patients during their visits to reinforce the importance of lifestyle modifications.

## Introduction

The current data on the prevalence of diabetes in Sri Lanka show an upward trend [1]. Diabetes mellitus is a major risk factor for cardiovascular diseases (CVDs) such as acute coronary syndrome, heart failure, and stroke. The risk of mortality from coronary heart disease and ischemic stroke increases two- to four-fold in patients with type 2 diabetes mellitus (T2DM) [2]. CVDs are reported to be the leading cause of diabetes-related mortality in Sri Lanka [3]. The increased risk of atherosclerotic CVD in people with T2DM is well-recognized, and the focus currently is on reducing cardiovascular risks as much as glycemic control. An individualized, multifactorial approach to treating type 2 diabetic patients is recommended, including lifestyle modifications and drug therapies with new diabetic agents, such as sodium-glucose cotransporter-2 (SGLT-2) inhibitors and glucagon-like peptide-1 (GLP-1) receptor agonists, to reduce the risk of CVD and improve renal outcomes, resting blood pressure control, and lipid and glucose levels [4, 5].

Only a few studies have been conducted to assess the risk factors for CVDs in the non-diabetic population in multiple locations in Sri Lanka. It has been observed that many of the admissions to the general medical wards at District General Hospital Kilinochi were due to diabetes. However, related studies of the cardiovascular risk factors among T2DM patients who received follow-ups in the clinics have been limited in Sri Lanka. Since diabetic patients are more likely to suffer from CVD than the rest of the population, increasing their knowledge and awareness of the disease and risk factors at the appropriate time can reduce their chances of developing complications related to their underlying condition, including morbidity and mortality. Accordingly, the present study aimed to assess the prevalence of additional risk factors for cardiovascular disease and awareness of these risk factors among diabetic patients attending a tertiary hospital.

## Materials and methods

Study design and place of study

This hospital-based, descriptive, cross-sectional study was conducted from June 2021 through August 2021. It involved patients with T2DM admitted to the general medical wards and attending medical clinics at the District General Hospital Kilinochchi.

Sampling

The patients were recruited through a systematic sampling of every third patient until the estimated sample size of 421 was reached. As no previous studies were available on this subject from the Kilinochchi district or Northern Province, we used the prevalence of risk factors from a previous study in Devinuwara, Matara District [6].

The following formula [7] was used for the sample size calculation of each risk factor, and the maximum sample size was taken as the minimal sample size for our study:

N=Z^2^ P(1-P)/ d^2^

N: Minimal sample size

Z: Critical value (1.96) of the specified CI, which is 95%.

P: Anticipated population proportion (See Table).

d: Absolute precision required on either side of the proportion (5%).

The minimum sample calculated for a family history of CVD (47%). A total of 383 was taken as a minimal sample size. Non-responsive rate of 10% is assumed. So the final sample inflated 421.

Inclusion criteria

All patients with T2DM, above 18 years, admitted to the general medical wards, and paid a visit to the medical clinic of District General Hospital Kilinochchi during the study period were recruited in this study.

Exclusion criteria

Patients who were seriously ill at the time of the interview, unable to provide information, suffering from type I diabetes mellitus or gestational diabetes, or mentally unstable were excluded from the sample.

Study instrument

The principal investigator conducted the interviews of the patients using the questionnaire and reviewed their medical records (paper-based clinic records) to collect further information such as HbA1C, last blood pressure measurements, cholesterol values, and so on. The questionnaire was prepared based on variables used in previous studies, especially for physical activity (International Physical Activity Questionnaire [IPAQ]), smoking, alcohol intake, and diet intake, which were validated already [8-10]. The study instrument was pre-tested and modified according to the responses and feedback from the PGIM reviewers and ethical review committee. Ethical clearance was obtained from the Ethical Review Committee for the Post Graduate Institute of Medicine at the University of Colombo, Sri Lanka (ERC/PGIM/2021/062).

Statistical analysis

The data were entered in an Excel sheet and then transferred and analyzed using SPSS version 28. The tables and figures present the prescriptive and summary statistics. The 95% CI was calculated for the additional risk factor percentages. The data were expressed as percentages, and bivariate analysis served to identify the socio-demographic variables associated with the selected risk factors using the Chi-squared test. P-values of less than 0.05 were considered statistically significant.

## Results

Background characteristics of the participants

Most of the 421 diabetic patients in this study (295, 70.1%) were women. The mean age of the participants was 57.36 ± 12.82 years, with a range of 20-89 years; a plurality belonged to the 41-60 age group (46.5%). Most (248, or 58.9%) had completed secondary school, a few (19, or 4.9%) had tertiary or above schooling, and a few others (11, or 2.6%) had not attended school. Most were married (76.6%) and living with their spouses at home (74.1%). One-third (33.3%) reported being currently employed, while a slightly smaller proportion (29.2%) were unemployed, and a few (2.6%) were not working because of disability. The working participants were involved in teaching (6.6%), office work (4.5%), farming (4.0%), other forms of labor (3.8%), business (2.8%), and transportation (2.4%). Most (81.6%) were in low-income households (<Rs 30,000); the median family income was Rs 16,000 (Table 1).

**Table 1:** Socio-demographic factors of the participants.

Socio-demographic Factors	Categories	Number	Percentage
Age	18-40	47	11.2%
	41-60	195	46.5%
	61-80	167	39.7%
	81 and above	12	2.9%
Gender	Male	126	29.9%
	Female	295	70.1%
Education	No Formal Education	11	2.6%
	Primary school	162	38.5%
	Secondary school	229	54.4%
	Tertiary/above	19	4.9%
Family income LKR	<10,000	153	36.3%
	10,001-30,000	191	45.3%
	30,001-50,000	65	15.4%
	50,001-100,000	10	2.4%
	>100,000	2	0.5%
Civil status	Married	322	76.5%
	Unmarried	8	1.9%
	Divorced	1	0.2%
	Separated	9	2.1%
	Widowed	81	19.2%
Employment status	Currently working	139	33.0%
	Retired	40	9.5%
	Unable to work because of disability	11	2.6%
	Homemaker	108	25.7%
	Unemployed	123	29.2%
Occupations of those working	Farmers	17	4.0%
	Garment worker	4	0.1%
	Driver	10	2.4%
	Teacher	28	6.6%
	Labourer	16	3.8%
	Office worker	19	4.5%
	Carpenter	2	0.05%
	Businessperson	12	2.8%
	Other	31	7.4%
Living status	Lives alone at home	14	3.3%
	Living with spouse at home	312	74.2%
	Living with children at home	82	19.5%
	Living with others at home	8	1.9%
	Living in elder care	5	1.2%
Ethnicity	Tamil	418	99.3%
	Sinhalese	1	0.2%
	Muslim	2	0.5%

Modifiable (lifestyle-related) cardiovascular risk factors

Most of the participants (83.6%) had a BMI in the normal range, while some (9.0%) were overweight, and a few others (2.1%) were obese. Fifty-two (12.2%) reported having smoked, while 37 (8.8%) were currently smoking and not ready to quit. Ten (2.4%) reported currently using alcohol. Around one-quarter (23.5%) engaged in too little physical activity, but most (73.4%) were moderately active. However, only around one-quarter (24.7%) met Sri Lanka's dietary guidelines regarding the consumption of fruits and vegetables (Table 2).

**Table 2:** Participants’ lifestyle-related CVD risk factors. CVD: Cardiovascular disease.

Lifestyle Risk Factors	Categories	Number	Percentage
BMI	Underweight (BMI< 18.5 kg/m^2 ^)	22	5.2%
	Normal (BMI 18.5-22.9 kg/m^2 ^)	352	83.6%
	Overweight (BMI-23-27.5 kg/m^2^)	38	9.0%
	Obese (BMI>27.5 kg/m^2 ^)	9	2.1%
Smoking status	Yes	52	12.2%
	Currently smoking	37	8.8%
	Ex-smoker	15	3.4%
	Never smoked	269	87.9%
Alcohol	Never drank	378	89.8%
	Ex-drinker	33	7.8%
	Current drinker	10	2.4%
	< or = 3 drinks per day	1	0.2%
	4-7 per drinks per day	1	0.2%
	7-14 drinks per week	4	1.0%
	15-21 drinks per week	4	1.0%
Physical activity	Insufficiently active	99	23.5%
	Moderately active	309	73.4%
	Highly active	13	3.1%
Met dietary guidelines	Yes	104	24.7%
	No	317	75.3%

The mean duration of T2DM among the participants in the study was 6.1 ± 4.1 years. The prevalence of major CVDs such as ischemic heart disease (IHD), stroke, or peripheral vascular disease (PVD) was 4.7% (CI: 11.6-18.4), (1.9%), and (0.4%), respectively. In addition, 14 patients (3.3%; 95% CI: 1.9-5.4) were found to have chronic kidney disease (CKD) based on the record, and 26 (6.2%) had microalbuminuria. Around half (49.4%; 95% CI: 44.6-54.2) of the participants were receiving treatment for hypertension. Hypercholesterolemia was found in 67.7% (95% CI: 63.1-72.0). Forty-two patients (10.2%; 95% CI: 7.4-13.1) reported a family history of CVD (Table 3).

**Table 3:** Participants’ established CVD risk factors. CVD: Cardiovascular disease.

Risk Factors	Number (%; 95% CI)
Family history of CVD	42 (10.2%: 7.4-13.1)
Past history of IHD	62 (14.7%: 11.6-18.4)
Past history of stroke	8 (1.9%: 0.9-3.6)
Past history of PVD	2 (0. 4%: 0.1-1.6)
Chronic kidney disease-based on records	14 (3.3%: 1.9-5.4)
Microalbuminuria	26 (6.2%: 4.2-8.8)
Elevated cholesterol level	285 (67.7%: 63.1-72.0)
Hypertension	208 (49.4%: 44.6-54.2)

Assessment of the patients’ awareness of the risk factors for CVD

All but two of the 421 patients (99.5%) reported having visited a doctor or a healthcare worker within the past year. Table 4 summarizes the patients' reporting of the advice they received from healthcare professionals. Among those who had a smoking history, most had been advised to quit or not to start smoking (24 of 37, or 64.9%). The majority of participants also received advice to reduce their intake of salt (87.9%) and sugary beverages (97.4%). However, only slightly more than half (53.0%) had received advice regarding the recommended servings of fruits or vegetables, and less than half had received advice about maintaining a healthy weight (40.8%) or engaging in physical activity (44.7%).

**Table 4:** Patients’ reporting of advice received from health professionals.

Advice	Response	Number	Percentage
Quit smoking	Yes	24	64.9%
	No	13	35.1%
Reduce salt intake	Yes	370	87.9%
	No	51	12.1%
Eat recommended servings of fruits/vegetables	Yes	223	53.0%
	No	198	42.0%
Reduce fat intake	Yes	337	80.0%
	No	84	20.0%
Increase physical	Yes	188	44.7%
	No	233	55.3%
Maintain a healthy weight	Yes	197	40.8%
	No	224	53.2%
Reduce sugar intake	Yes	410	97.4%
	No	11	2.6%

The BP of all of the patients had been tested by a doctor or healthcare worker, but only 208 (49.4%) had been informed about high BP. In addition, the blood cholesterol levels of most (94.5%) were checked. Three hundred and twelve patients (74.1%) were being treated with statins for either primary or secondary prevention of CVD, but only 285 (67.7%) were aware that they were receiving statin therapy. One hundred and sixty-three patients (38.7%) were on aspirin therapy, all of whom were aware of the fact and showed good compliance (Table 5).

**Table 5:** Assessment of participants’ awareness of selected factors.

Questions	Responses N (%)	
	Yes	No
1. Have you visited a doctor or healthcare worker during the past 12 months?	419 (99.5%)	2 (0.5%)
2. Have you been told by a doctor or healthcare worker that you have high BP/hypertension?	208 (49.4%)	213 (50.6%)
3. Has your cholesterol been measured?	398 (94.5%)	23 (5.5%)
4. Have you ever been told by a doctor or healthcare worker that you have elevated levels of cholesterol?	285 (67.7%)	136 (32.3%)
5. Are you currently taking statins (e.g., atorvastatin/rosuvastatin) to treat or prevent CVD?	312 (74.1%)	109 (25.9%)
6. Are you regularly taking aspirin to treat or prevent CVD?	163 (38.7%)	258 (61.3%)
6. Is your diabetes mellitus controlled?	373 (87.6%)	48 (11.4%)
7. Are you aware of your last HbA1C?	35 (8.3%)	386 (91.7%)

The diastolic blood pressure (DBP) of 226 patients (53.2%) was within the target range, while only 68 (16.2%) were within the target range for systolic blood pressure (SBP). Most of those with hypertension were being treated with an angiotensin receptor blocker (ARB) (22.8%) and an angiotensin-converting enzyme inhibitor (ACE-I) (11.9%). Two hundred and eight of the patients had hypertension, but only 158 (76%) were receiving antihypertensive medication. In addition, most of the patients (66.5%) had total cholesterol readings within the target range, but less than half were within the target range for LDL (40.1%) or triglycerides (39.9%).

**Figure 1:**
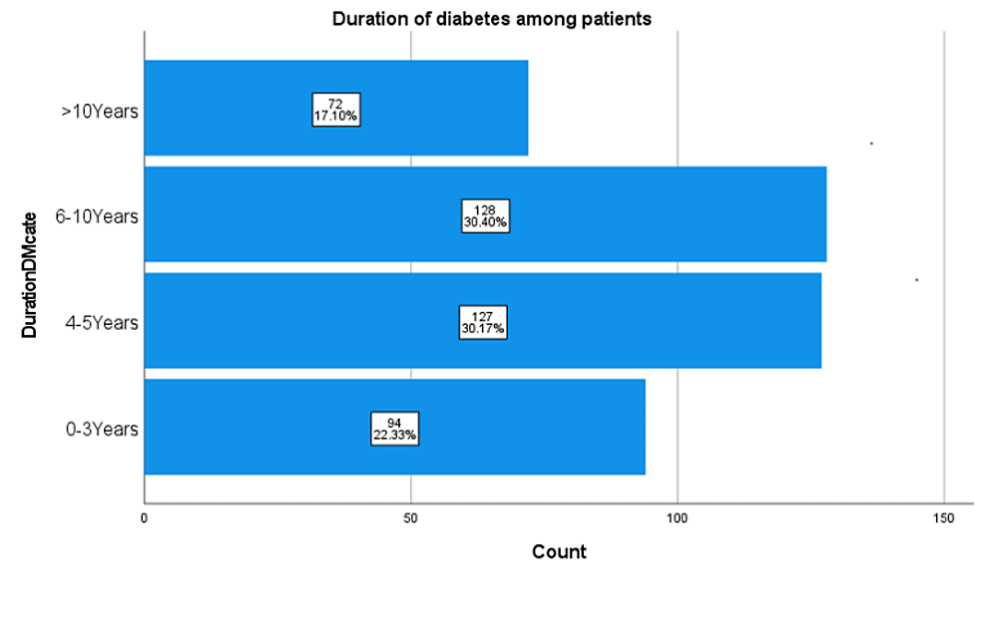
Duration of diabetes among patients.

This research also took into account current diabetic management at the time of interviews. A roughly equal percentage of patients (30.2%) had diabetes for around 4-5 years and 6-10 years (30.4%), as Figure 1 shows. A smaller percentage (17.1%) had had diabetes for over 10 years. The mean duration was 6.16 ± 4.1 years. T2DM was controlled (HbA1C<7) in most of the patients (373, or 88.6%), though nearly all (91.7%) were unaware of their most recent HbA1C level measurement. The mean HbA1C of study participants was 7.64±1.23. Notably, most had been treated with metformin (395, or 93.8%) and/or a sulphonylurea (296, or 70.3%). Smaller numbers were on metformin (63, or 15.0%) or sulfonylureas alone (5, or 1.2%). Among 61 patients (14.5) who were on insulin, 14 (3.3%) were on insulin alone.

Of the socio-demographic factors analyzed for their influence on diabetic control, only civil status (also referred to as marital status) had a significant association (P-0.037). Neither gender, educational status, age, income, nor employment status was significantly associated with diabetic control (P>0.05).

Adherence to aspirin and statin therapies

According to their records, 409 patients were prescribed aspirin (97.1%), but only 163 took it (39.8%). Similarly, 415 patients (98.5%) were prescribed statins, but only 312 were taking the statins (75.2%).

Influence of demographic factors on lifestyle-related CVD among diabetic patients

Table 6 summarizes the analysis of the influence of socio-demographic factors such as age, gender, income, educational level, employment status, and marital status on selected modifiable risk factors of CVD among diabetic patients.

**Table 6:** Association of socio-demographic factors and lifestyle-related risk factors.

Socio-demographic variable	Lifestyle-related cardiovascular risk factors			
	Smoking status	Dietary guidelines for fruit and vegetable consumption	Physical activity	BMI
	P-value			
Gender	P<0.001	P-0.052	P<0.001	P-0.064
Education	P-0.947	P-0.004	P-0.002	P<0.001
Civil status	P-0.698	P-0.322	P<0.001	P-0.014
Age category	P<0.001	P-0.151	P<0.001	P-0.004
Working status	P<0.001	P-0.053	P<0.001	P-0.001
Family income	P-0.76	P-0.096	P-0.052	P-0.072

The patient's smoking status differed significantly by gender (P<0.001), age (P<0.001), and working status (P<0.001) but not by family income, civil status, or educational status (P>0.05). Fifty male patients (39.7%) were current or former smokers, but only two (0.5%) of the female patients were current or former smokers. Similarly, smoking was more common among patients over the age of 60 (36.5%) than in the under-60 age group (63.5%). Only 2 (0.5%) of currently working patients reported smoking compared with 13 (3.1%) of the unemployed patients, while 12 (2.8%) of the retired patients reported smoking.

Among the socio-demographic factors, only educational status being significantly associated with adherence to the recommended dietary guidelines for the consumption of vegetables and fruit (P-0.004). The likelihood of engaging in moderate physical activity was significantly associated with gender (P<0.001), educational status (P-0.002), civil status (P<0.001), age (P<0.001), and working status (P-0.001). Likewise, educational status (P<0.001), civil status (P-0.014), working status (P-0.001), and age (P-0.004) were significantly negatively associated with being overweight or obese.

## Discussion

T2DM is often associated with microvascular and macrovascular complications, including retinopathy, neuropathy, nephropathy, IHD, stroke, and PVD. In addition, CVD remains a leading cause of death among diabetes patients [2]. Traditionally, the management of diabetes has focused on the strict control of blood glucose. However, recent findings demonstrate that treating other cardiovascular risk factors is equally important for reducing mortality and morbidity among these patients. Therefore, researchers have advocated lifestyle modifications that enhance control of blood pressure and lipid and glycemic levels in order to improve the outcomes of CVD along with diabetic drugs such as GLP-1 receptor agonists and SGLT-2 inhibitors in recent years [5].

Among the 421 diabetic patients in this study, 70.1% were women. The reason females were common among diabetic patients could be because 30 years of the civil war left this district with a female-headed family, and stress could be a contributing factor that needs further investigation. Saeedi P et al. [11] found the pooled prevalence rates to be 9.9% (95% CI, 8.8-11.0%) among men and 11.6% (95% CI, 10.0-13.1%) among women. Further, in the present study, 46.5% of the patients (195) belonged to the 40-60 age group, with a mean age of 56.7. However, the age of diagnosis has been decreasing gradually, from 48.5 years in 1995 to 36.14 in 2016-2018, as Patel B et al. [12] noted in a previous study.

Obesity is commonly associated with diabetes and represents a common link between two possible forms of low-grade inflammation [13]. Similarly, Gamlath L et al. [14] reported that 56% of diabetic patients in Sri Lanka were obese. In our study population, though, only 2.1% of the patients were obese, and another 9.0% were overweight. This lack of consistency in the findings may be due to the fact that the region experienced 30 years of civil war that resulted in undernutrition. In like manner, the large portion (75.3%) of the patients in this study who did not comply with the Sri Lankan dietary guidelines for vegetable and fruit consumption may be due to limited purchasing ability. A world health survey found that, in low- and middle-income countries, 77.6% of men and 78.4% of women consumed less than the minimum recommended level of five servings of fruits and vegetables per day [15]. Further, in the present study, 23.5% of the patients reported engaging in insufficient physical activity-based IPAQ. A meta-analysis by Kraus WE et al. [16] found 150 minutes of moderate-intensity aerobic activity weekly to be associated with reductions in CVD mortality by 23% and in the incidence of T2DM by 26%. Active smoking is significantly associated with an increased risk of cardiovascular events and mortality among diabetes patients. A systematic review and meta-analysis of cohort studies by Sarwar N et al. [2] found the pooled relative risk (95% CI) of total CVD to be 1.44 (1.34-1.54). Twenty percent of the participants in the present study were either former or current smokers, and none of the latter were ready to quit. Gamlath L et al. [14] reported that 8% of diabetics in Sri Lanka smoked, and the same percentage consumed alcohol. In the present study, only 2.4% of the patients reported consuming alcohol.

Also, in the present study, 218 patients (49.4%; CI: 44.6-54.2) received treatment for hypertension. In another study conducted in Sri Lanka, Rathnayake RK et al. [6] found a higher prevalence of hypertension among diabetes patients (67%). Hypertension and diabetes usually co-exist, and diabetes independently contributes to the development of atherosclerosis, but hypertension increases the incidence and progression of cardiovascular events [17]. Diabetes has also been significantly associated with visit-to-visit variability in blood pressure measurements [18]. Many diabetic guidelines recommend SBP and DBP targets below 140 and 90 mm Hg, respectively, but below 130 and 80 mm Hg when significant proteinuria exists [19, 20]. Therefore, tight blood pressure control seems essential for preventing cardiovascular events. In the present study, 226 patients (53.2%) were in the target range for DBP, but only 68 (16.2%) were within the target range for SBP. BP is a significant area of concern, and healthcare workers need to be educated regarding these findings. ACE-I or ARB is the treatment of choice for hypertension with co-existing diabetes [19, 20], and most patients in the present study (92.4%) received the appropriate treatment. Few interventional studies of salt restriction among diabetes patients are available. However, most guidelines recommend limiting the intake of dietary salt. Doing so has been found to correlate with lower BP levels in the general population and lower incidences of cardiovascular events and all-cause mortality possibly associated with "water retention, increase in systemic peripheral resistance, alterations in the endothelial function, changes in the structure and function of large elastic arteries, modification in sympathetic activity, and in the autonomic neuronal modulation of the cardiovascular system" [21]. Nearly all of the participants in the present study (95.5%) were aware that salt in their diet could cause health problems, but few were trying to reduce their intake of it.

Nearly all participants (99.5%) had likewise visited a doctor or healthcare professional within the previous year. In addition, most had received advice on reducing their intake of salt (87.9) and sugary beverages (97.4%). However, in a global survey of the knowledge and awareness of CVD risk factors in people with T2DM by Saeedi P et al. [22], one in six respondents (17%) had never discussed their health risks with a healthcare professional. One in eleven (9%) was unaware of CVD and the associated risk factors.

Assessing CVD risk, establishing lipid targets, and using statin or other therapies are essential steps in reducing the frequency of cardiovascular events among patients with T2DB and dyslipidemia. Insulin resistance in peripheral adipose tissue and the liver is possible pathophysiology associated with dyslipidemia [4]. This study found hypercholesterolemia in 67.7% of the patients (95% CI: 63.1-72.0), while Rathnayake RK et al. [6] reported 80%. Statins have proved effective in reducing LDL cholesterol, but Parris ES et al. [23] reported that patients with dyslipidemia, including those with diabetes, commonly failed to reach their LDL cholesterol targets. In the present study, 75.2% of the participants were adhering to their statin therapy. Though the total cholesterol of 66.5% of the patients was within the target range, the LDL cholesterol of only 40.1% was within the target range. This could be due to physicians' inertia in regard to intensifying statin therapy.

Further, most of the CVDs experienced by the patients were in the form of IHD, stroke, and PVD, the numbers being 62 (14.7% 95% CI: 11.6-18.4), 8 (1.9%), and 2 (0.4%), respectively. A study that Rathnayake RK et al. [6] conducted at the National Hospital of Sri Lanka found a similar prevalence of IHD, stroke, and PVD at 10.6%, 1.1%, and 4.7%, respectively. Further, 14 patients (3.3%; 95% CI: 1.9-5.4) were found to have CKD and 26 (6.2%) to have microalbuminuria (timed overnight collection: above 20 mcg/min or spot collection above 20 mg/L). According to a review of records, 409 patients (97.1%) were prescribed aspirin. Aspirin has an established role in the secondary prevention of CVD [24], but its use as primary prevention for diabetes is questionable [24-27]. In any case, only 39.8% of the patients adhered to aspirin therapy. There is, therefore, an apparent need to review the use of aspirin therapy in this context.

Recently, many cardiologists have begun to advocate the use of new diabetes medications, such as glucagon-like peptide-1 (GLP-1) agonists and sodium-glucose cotransporter-2 (SGLT-2) inhibitors [28-30]. In our study, T2DM was controlled in most of the patients (88.6%), and most (93.8%) were being treated with metformin and/or a sulphonylurea (296; 70.3%). However, only six patients (1.4%) were prescribed either GLP-1 agonists or SGLT-2, possibly because these medications are unavailable in the public hospital system.

Lastly, socio-demographic factors such as gender, age, and working status are significantly associated with smoking status and physical activity, and similarly, educational status significantly influenced the likelihood of compliance with the recommended consumption of vegetables and fruits, thus need to be considered when educating diabetes patients regarding cardiovascular risks. 

Limitations

Since the questionnaire was administered in face-to-face interviews and the primary data collection was done by the principal investigator, there may have been a tendency for the study subjects to hide certain information during their interviews, especially with regard to the assessment of practice. Further samples were not stratified between inward and outpatient clinics. Further population-based studies with a large sample would have improved the quality of the data presented here. Also, as the study participants of this study were from a single district of Sri Lanka, generalizing the findings needs caution even though this study reported a significant issue from a region affected by 30 years of civil war.

## Conclusions

Lifestyle modifications, good glycemic and blood pressure control, and treatment of dyslipidemia reduce the CVD risks associated with diabetes. This study has revealed similar gaps as in the literature related to lifestyle modifications and recommended practices for reducing CVD risks. A significant portion of the patients in this study had uncontrolled DBP, and the SBP of most was not within the target range. Similarly, the LDL cholesterol of a significant portion of the patients was not within the target range. Accordingly, a review of lipid management is recommended, along with efforts to address physicians' inertia. Similarly, since the patients' adherence to aspirin therapy was poor, education in this regard is essential during routine visits and reinforcement of lifestyle modifications. These findings are significant for middle-income countries and those affected by a long-standing war.
